# Psoriatic arthritis: review of potential biomarkers predicting response to TNF inhibitors

**DOI:** 10.1007/s10787-022-01092-x

**Published:** 2022-12-12

**Authors:** Anaïs Makos, J. H. Kuiper, O. Kehoe, R. Amarasena

**Affiliations:** 1 School of Medicine, Keele University, at the RJAH Orthopaedic Hospital, Oswestry, SY10 7AG UK; 2 School of Pharmacy & Bioengineering, Keele University, at the RJAH Orthopaedic Hospital, Oswestry, SY10 7AG UK; 3 Robert Jones and Agnes Hunt (RJAH) Orthopaedic Hospital, Oswestry, SY10 7AG UK

**Keywords:** Psoriatic arthritis, Biologics, Tumor necrosis factor inhibitor, Biomarkers, Resistance

## Abstract

Psoriatic arthritis (PsA) is a chronic and painful inflammatory immune-mediated disease. It affects up to 40% of people with psoriasis and it is associated with several comorbidities such as obesity, diabetes, metabolic syndrome, and hypertension. PsA is difficult to diagnose because of its diverse symptoms, namely axial and peripheral arthritis, enthesitis, dactylitis, skin changes, and nail dystrophy. Different drugs exist to treat the inflammation and pain. When patients do not respond to conventional drugs, they are treated with biologic drugs. Tumour necrosis factor inhibitors (TNFi’s) are commonly given as the first biologic drug; beside being expensive, they also lack efficacy in 50% of patients. A biomarker predicting individual patient’s response to TNFi would help treating them earlier with an appropriate biologic drug. This study aimed to review the literature to identify potential biomarkers that should be investigated for their predictive ability. Several such biomarkers were identified, namely transmembrane TNFα (tmTNF), human serum albumin (HSA) and its half-life receptor, the neonatal Fc receptor (FcRn) which is also involved in IgG lifespan; calprotectin, high mobility group protein B1 (HMGB1) and advanced glycation end products (AGEs) whose overexpression lead to excessive production of pro-inflammatory cytokines; lymphotoxin α (LTα) which induces inflammation by binding to TNF receptor (TNFR); and T helper 17 (Th17) cells which induce inflammation by IL-17A secretion.

## Introduction

Psoriatic arthritis (PsA) is a heterogeneous chronic immune-mediated inflammatory joint disease. Multiple characteristics define this disease, such as arthritis of the spine and limbs (axial and peripheral arthritis), inflammation where tendons or ligaments are joined to bone (enthesitis), swelling of fingers or toes (dactylitis), and skin and nail changes. Symptoms can be found in isolation or in combination with one another (Gottlieb and Merola 2021). In most cases, the disease occurs in association with a skin disorder known as psoriasis. Psoriasis affects 1–3% of the white population, and arthritis occurs in 10–40% of psoriasis patients (Ogdie and Weiss 2015).

No curative treatments of PsA are available, and the choice of treatment mainly depends on efficacy, safety, and cost. Currently, rheumatologists follow the EULAR recommendations to manage PsA patients. Usually, non-steroidal anti-inflammatory drugs (NSAIDs) such as naproxen, diclofenac or celecoxib are prescribed first, often combined with local injection of glucocorticoids. The next step is administration of conventional synthetic disease-modifying anti-rheumatic drugs (csDMARDs), first as single drug and if ineffective in combination. Common csDMARDs are methotrexate (MTX), leflunomide (LEF), and sulfasalazine (SSZ). Biologic therapies are used when patients fail to respond to csDMARDs. These are commonly divided into four distinct groups: (1) TNF inhibitors (TNFi) such as adalimumab (ADA), etanercept (ETA), golimumab (GOL), certolizumab pegol (CET) and infliximab (INF), (2) IL-12/23 inhibitors (IL-23i) such as ustekinumab (UST) and guselkumab (GUS), (3) IL-17 inhibitors (IL-17i) such as secukinumab (SEC) and ixekizumab (IXE), and (4) Janus kinase/signal transducer and activator of transcription inhibitor (JAK/STATi) such as tofacitinib (TOF) (Kamata and Tada 2020; Ogdie et al. 2020; Zhang et al. 2021; Chen and Dai 2020). Further biologics exist in the form of targeted oral agents, including phosphodiesterase-4 inhibitors (PDE-4i) such as apremilast (APR) which are used when other biologics are contraindicated (Ogdie et al. 2020).

Unfortunately, many patients do not respond to biological drugs, with at least 40% of patients partially responding or failing to respond to biologics (Veale and Fearon 2018). TNFi is often the first prescribed class of bDMARD; although TNFi is well tolerated, it is ineffective in up to only 40% of patients followed on a 2-year period, and may be ineffective in up to 50% of patients for long-term therapy (Clunie et al. 2018). A biomarker easily detected in peripheral blood samples, able to identify PsA patients who do not respond to TNFi, could help in the choice of a first biologic treatment. The objective of this review is to find and highlight candidates for such biomarkers and to explain how they could predict treatment resistance.

## Method

Using the PubMed database, we reviewed articles published from 1973 (first description of PsA by Moll and Wright) to 2022. We used a combination of the keywords “psoriatic arthritis”, “TNF inhibitor”, “biomarkers”, “failure”, and “response”. We screened abstracts and read the relevant articles, and short-listed articles were mostly published between 2005 and 2022. References of relevant articles were also screened and read if appearing to be relevant. All types of articles were included (literature reviews, observational studies, reports of clinical trials and meta-analysis). If potential biomarkers became recurrent in our reading, we focused our literature research on these molecules and their receptors, and on the immune cells secreting them. This is a non-systematic review and there were no formal inclusion or exclusion criteria.

## Results

### TNFα and its receptors

TNFα is a pro-inflammatory cytokine produced mainly by macrophages and monocytes. Many other cells can also produce this cytokine, such as B and T cells, natural killer (NK) cells, dendritic cells (DCs), neutrophils, mast cells, keratinocytes, endothelial cells, smooth muscle cells, cardiomyocytes, fibroblasts, osteoblasts and osteoclasts, adipocytes, astrocytes, microglial cells, adrenocortical cells, and glomerular mesangial cells (Bradley 2008; Lin et al. 2000). TNFα can be produced in two different forms, soluble TNFα (sTNF) and transmembrane TNFα (tmTNF). They both work as active homodimers and have different biological activities, with tmTNF more active than sTNF (Zelová and Hošek 2013) when binding to their receptor TNFR comprising an extracellular domain that forms the ligand-binding domain, a transmembrane domain, and an intracellular domain that interacts with proteins in the cytosol to induce signalling (Zelová and Hošek 2013). Two different TNFRs bind TNFα, TNFR1, and TNFR2. TNFR1 is expressed at the surface of all cell type except erythrocytes. tmTNF and sTNF can both activate TNFR1. Depending on the protein adaptor involved in the signalling complex, the binding stimulates expression of either pro-inflammatory and cell survival genes, or apoptosis and cell death genes.

TNFα play a major and pivotal role in the development of joint inflammation (Mease et al. 2000) and skin psoriasis via keratinocyte proliferation and induction of plaque psoriasis (Giustizieri et al. 2001). Indeed, TNFα activates naive CD4 + T cells, in association with other pro-inflammatory cytokines such as IL-6 and IL-1. Once activated, these cells that are present in large amount at sites of inflammation will produce more pro-inflammatory cytokines, including TNFα. TNFα can enhance the inflammatory response via osteoclast activation, leading to osteoclastogenesis, bone resorption, and joint erosion and destruction. TNFα can also be produced by T helper (Th17) cells. In association with other pro-inflammatory cytokines, it can promote the proliferation of keratinocytes and reduce their differentiation to induce skin inflammation and plaque psoriasis (Giustizieri et al. 2001); Prieto-Pérez et al. 2013).

### TNFi in PsA

**Fig. 1:**
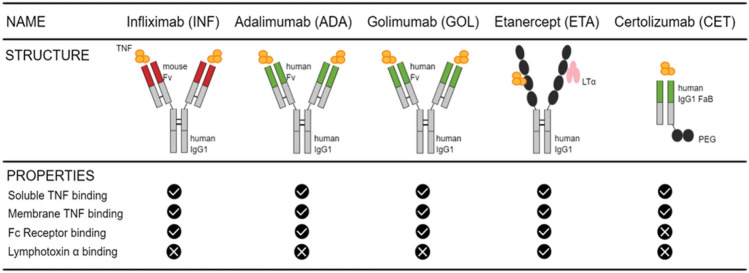
Structure of the common TNFi and their binding properties. Common TNFi administered to treat PsA are infliximab, adalimumab, golimumab, etanercept, and certolizumab. The schematic structure of each Ab is represented as well as their capacity to bind sTNF and tmTNF, FcR and LTα. Modified from Sedger and McDermott (2014)

Because TNFα plays such an important role in PsA pathogenesis, TNFi are the first-line biologics used to treat the disease when patients fail to respond to csDMARDs. Moreover, TNFi are less expensive than most recently developed biologics (Information et al. 2017). They are administered mainly to reduce the inflammation induced by TNFα. Commonly used TNFi to treat PsA are the following: infliximab (INF), adalimumab (ADA), golimumab (GOL), certolizumab pegol (CET), and etanercept (ETA) (Sedger and McDermott 2014) (Fig. 1). All five are human TNF-specific neutralizing antibodies binding to sTNFα and tmTNFα and thereby inhibiting binding to TNFR, thus inhibiting signal transduction effectively stopping the biologic activities of TNFα (Winterfield and Menter 2004). INF is a monoclonal chimeric human–mouse antibody (Ab) that was approved by the Food and Drug Administration (FDA) in 2005 to treat PsA (Ducharme and Weinberg 2008). It is composed of a complement-fixing human immunoglobulin (Ig) G1 (IgG1) constant region (75%) and a murine-derived antigen-binding variable region (25%) (Liang et al. 2013). INF has two antigen-binding surfaces, so one Ab can bind two molecules of TNFα, giving a stable binding (Winterfield and Menter 2004). ADA and GOL are fully humanized IgG1 anti-TNF with an Fc fragment identical to INF and an engineered human fragment variable (Fv) sequence for the fragment antigen-binding (Fab) fragment (Sedger and McDermott 2014). They were approved by the FDA to treat PsA in 2005 and 2009 (Ducharme and Weinberg 2008). CET is a PEGylated (polyethylene glycol or PEG) dimeric Ig Fab domain of a humanized TNF-specific IgG1 monoclonal antibody and was approved by the FDA in 2009 to treat PsA (Chimenti et al. 2013; Love and Kavanaugh 2018). The PEG increases the half-life of the drug allowing more lasting effect (Sedger and McDermott 2014). ETA is a fully human recombinant fusion protein and consists of an extracellular region of human TNFR2 expressed as a fusion protein with a C-terminal part of a human IgG1 crystallized fragment (Fc fragment) (Sedger and McDermott 2014; Anandarajah and Ritchlin 2003). It was approved in 2002 by the FDA to treat PsA (Ducharme and Weinberg 2008). INF, ADA, GOL, and ETA can bind to Fc receptors (FcRs). FcRs belong to the immunoreceptor tyrosine-based activation motifs (ITAM)-associated receptor family (Ben Mkaddem et al. 2019). FcRs include many receptors such as FcγRs, FcεRs, FcαRs, FcμRs, and the neonatal Fc Receptor (FcRn) (Li and Kimberly 2014). They play a role in humoral and innate immunity, and consequently in inflammatory and auto-immune diseases (Ben Mkaddem et al. 2019). Besides FcRs, ETA can also bind lymphotoxin α (LTα), a unique ability among TNFi. LTα is a natural ligand for TNFR able to promote inflammation when it binds to its receptor (Cuff et al. 1998).

### Candidate biomarkers of poor response to TNFα inhibitors

#### Candidate blood biomarkers

Potential blood biomarkers can be divided into four groups: (1) TNFR-related biomarkers including sTNF, tmTNF, the TNFα converting enzyme (TACE) and LTα, (2) half-life-related biomarkers, including FcRn and human serum albumin (HSA), (3) alarmin and inflammation biomarkers, including S100A8/A9 (calprotectin), high mobility group protein B1 (HNGB1), and advanced glycation end product (AGEs), and (4) cell component as a biomarker, including Th17 cells and regulatory T (Treg) cells. These biomarkers are easily detectable in serum and plasma samples with classical enzyme-linked immunosorbent assays (ELISAs). Peripheral blood mononuclear cells (PBMCs) can also be isolated from blood samples and analysed using western blot (WB), retro transcription quantitative polymerase chain reaction (RT-qPCR), and flow cytometry (Cecchinato et al. 2018).

##### Transmembrane versus soluble TNFα

TNFα can be found in two forms: tmTNF and sTNF. tmTNF is cleaved by TACE to create sTNF that enables induction of inflammation at sites distant from TNFα-producing cells. (Horiuchi et al. 2010). TNFi has been engineered to bind sTNF and tmTNF, preventing their binding to TNFR. Atreya and colleagues worked with patients with Crohn’s diseases treated with ADA, and used confocal laser endomicroscopy to analyze the number of tmTNF + cells after 12 week of treatment (Atreya et al. 2014). They found that patients with higher number of tmTNF + cells had significantly higher short-term response rates compared to patients with lower number of tmTNF + cells (response rate 92% vs 15%).

Interestingly, TNFi are used to reduce inflammation in both Crohn’s disease and PsA. Moreover, Crohn’s disease is one of the many comorbidities of PsA. We can suppose that if the level of tmTNF + cells impacts the response to treatment of patients with Crohn’s disease, it might also be involved in PsA patients’ response to TNFi. Consequently, PsA patients with a high level of tmTNF + cells might better respond to TNFi than patients with a low level of TNF + cells, and it may be used as a potential biomarker of response to treatment.

##### Human serum albumin (HSA) and its half-life regulator FcRn

HSA is the most abundant protein in plasma (Fanali et al. 2012). HSA has strong binding properties: in serum it can bind to metals, fatty acids, hormones, bilirubin, bile acid, but also to some drugs (Soeters et al. 2019; Nilsen et al. 2020). Due to these properties, HSA is studied for its use as a delivery carrier to affect drug pharmacokinetics. Ademowo and colleagues found that higher level of HSA could be one of the most important predictive biomarkers of a positive response to TNFi (Ademowo et al. 2016). Their finding was supported by Veering et al. who suggested that a lower concentration of HSA could decrease the binding of drug to TNFα (Veering et al. 1990). Hypoalbuminemia, is common in people with PsA and with inflammatory conditions in general, but also in people with comorbidities such as psoriasis, obesity, metabolic syndrome, and insulin resistance (Sheikh et al. 2015; Mosli and Mosli 2017; Soeters et al. 2019). In most cases, hypoalbuminemia is not due to a decreased synthesis because its fractional synthesis rate (FSR) in plasma is normal or even mildly increased in these patients. The more plausible explanation for low HSA levels is a shortened half-life and its inscape into the interstitial space due to increased permeability of the capillaries (Soeters et al. 2019). In the interstitial, increased breakdown of albumin occurs providing a source of amino acids and energy for cells (Soeters et al. 2019). Low HSA levels seem to be associated with a reduced TNFi concentration (Kopylov and Seidman 2016). Arias and colleagues worked on ulcerative colitis (UC) treated with INF. They showed that a low level of albumin was correlated to an increased clearance of INF in patients with UC (Arias et al. 2015). Moreover, Fasanmade and colleagues found that UC patients with higher levels of HSA maintained higher concentrations, lower clearance, and longer half-life of INF (Fasanmade et al. 2010). In patients who had normal ranges of HSA concentrations, clinical response to INF did not diminish because drug concentrations remained at therapeutic level. Lower HSA concentrations and lower serum INF concentrations were associated with a decreased response to the drug. No clear mechanism has been proposed to explain this phenomenon, but it has been hypothesized that FcRn may be responsible for the relationship between serum albumin and serum INF level (Fasanmade et al. 2010). INF is an engineered IgG, and FcRn plays a role in protecting both albumin and IgG from catabolism.

FcRn belongs to the family of Fc gamma receptor (FcγR). It can be found in intestine, epithelium, placenta, kidney, and liver, but also in cells of hematopoietic origin (monocytes, macrophages, neutrophils, DCs, and B cells). FcRn is mainly localized intracellularly and can bind HSA and IgG and save them from degradation via a recycling mechanism. FcRn, thus, regulates their half-life, and consequently their concentration in serum (Pyzik et al. 2019). The binding occurs in an acid environment via endocytosis of IgG or albumin in the cytoplasmic tail of FcRn, after which the complex is redirected from the lysosomal pathway to the plasma membrane. When pH increases and returns to physiologic levels, HSA or IgG dissociates from FcRn and are either recycled or transported away from the lysosome via transcytosis (Kuo et al. 2010; Stapleton et al. 2019). The five TNFi most commonly administered to patients with PsA possess in their structure a Fc fragment from human IgG and can bind FcR (Sedger and McDermott 2014). Consequently, these TNFi could also bind to FcRn, increasing their half-life and time in the blood circulation. The hypothesis is that a low HSA level caused by a decreased FcRn expression, could be a potential indicator for a low response to TNFα inhibitors, as the drug would be degraded a few days after injection.

##### Alarmins

Alarmins are constitutively expressed endogenous chemotactic and immune-activating molecules (Yang et al. 2017). They are also known as danger signals and are a subset of damage-associated molecular patterns (DAMPs) that interact with pattern recognition receptors (PRRs), in particular toll-like receptors (TLRs) and receptor of advanced glycation end products (RAGE).

TLRs can drive inflammation through production of pro-inflammatory cytokines after cell injury and infection. They can be cytosolic and/or membrane receptors, and are activated by DAMP and pathogen-associated molecular pattern (PAMP) binding (McCormack et al. 2009). Immune cells such as macrophages/monocytes, DCs, natural killer (NK) cells, mast cells, and granulocytes (basophils, neutrophils, and eosinophils) express TLRs on their surface (El-Zayat et al. 2019; Candia et al. 2007), as well as keratinocytes where they play a role in psoriatic skin (Sun et al. 2019). RAGE belongs to the immunoglobulin superfamily of cell surface molecules and is membrane bound (Mulrennan et al. 2015). It is expressed at the surface of endothelial cells and immune cells such as macrophages/monocytes, neutrophils, DCs, and B and T lymphocytes (Mulrennan et al. 2015; Kierdorf and Fritz 2013). Research on TLRs and RAGEs in PsA is limited but increasing expression of TLR2, 3, and 4 has been observed in synovial tissues (ST) and SF of patients with rheumatoid arthritis (RA) (Ospelt et al. 2008; Huang et al. 2007). Moreover, Candia and colleagues have found increased expression of TLR2 in immature DCs in patients with PsA (Candia et al. 2007). As for RAGE, it has been found in the SF of patients with RA and osteoarthritis (OA) (Chuah et al. 2013; Drinda et al. 2005), but no studies of patients with PsA were found. The ligand–receptor binding activate the innate immune system, enhancing the antigen-specific adaptive immunity (Sun et al. 2019), and inducing a signalling cascade leading to immune responses and inflammation (Nefla et al. 2016).

**Fig. 2:**
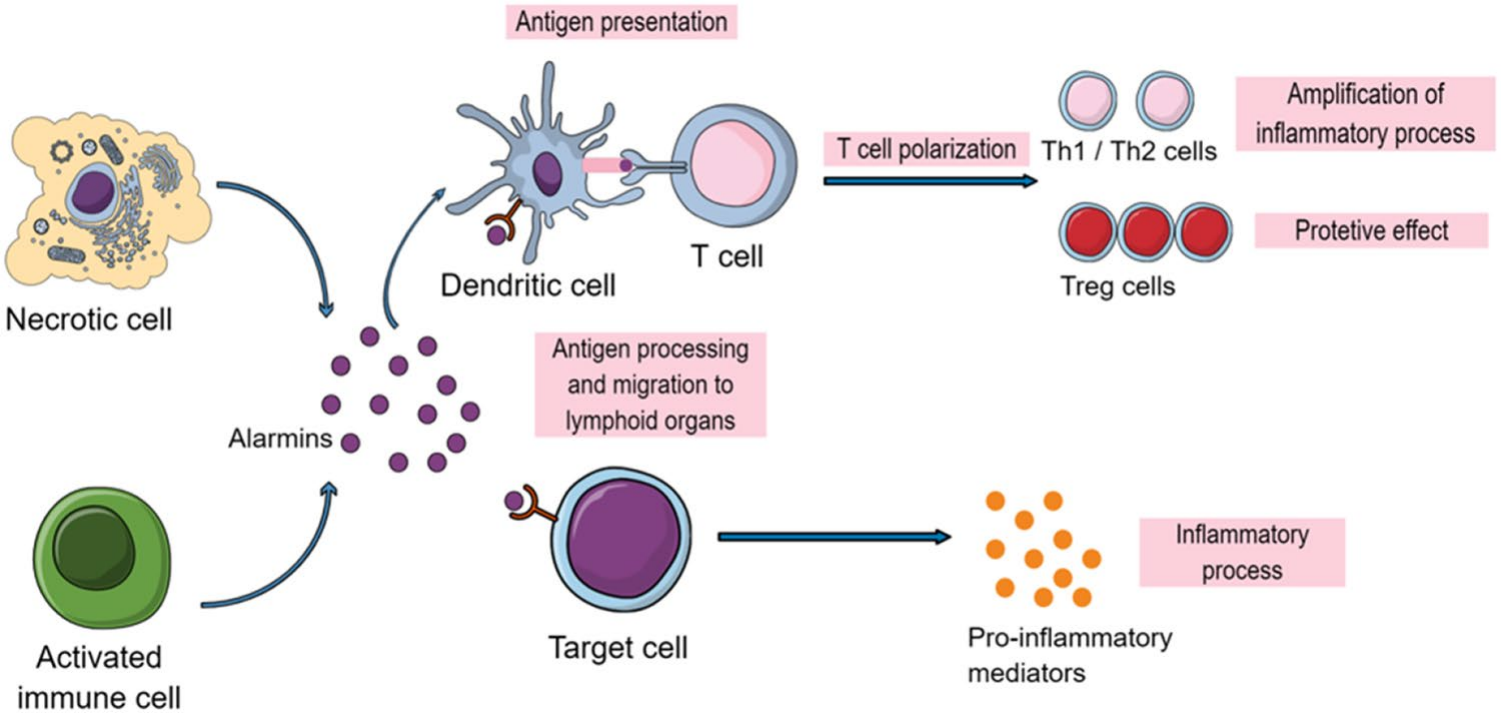
Alarmin’s sources and targets. Alarmins are danger molecules actively secreted by activated immune cells or passively released by necrotic cells. They can bind to their receptor at the surface of cells (immune cells and endothelial cells mainly) to induce the production of pro-inflammatory cytokines and enhance the inflammatory process. They can also recruit immature DCs so they can present antigen to T cells, leading to their polarization to either amplify the inflammatory process or have protective effects. (Modified from Nefla et al. (2016)

Alarmins have different origins and can be granule derived, nuclear or cytoplasmic (Yang et al. 2017). They can be passively released by necrotic cells or actively secreted by different types of cells such as neurons, enterocytes, smooth muscle cells, endothelial cells, epithelial cells but also immune cells such as myeloid and NK cells or phagocytes (Nefla et al. 2016; Bianchi 2007) (see Fig. 2).

Some alarmins are known to play a role in arthritis and inflammatory diseases, and have been studied in peripheral blood and SF from patients with RA (Biscetti et al. 2017; Nys et al. 2019), OA (Denoble et al. 2011; Ke et al. 2015) (Wei et al. 2013) and PsA (Aochi et al. 2011; Kane et al. 2003) as potential biomarkers of the diseases. S100A8/A9 and HMGB1 seem to play a role in inflammation in PsA patients.

*S100A8/A9* The S100A8/A9 proteins, also known as calgranulin A and B or as myeloid-related protein (MRP) 8/14, belong to the calcium-binding S100 protein family (Perera et al. 2010). S100A8 is principally secreted by keratinocytes and mononuclear cells (Wang et al. 2018) such as monocytes (Yang et al. 2018), whereas S100A9 is mainly secreted by neutrophils and keratinocytes and seem to protect skin and joints from chronic inflammation (Schenten et al. 2018; Mellor et al. 2022). Both can be found as homodimers or heterodimers. The latter named calprotectin has been found to be overexpressed in serum, SF and psoriatic plaque in patients with PsA (Wang et al. 2018). Calprotectin can bind to TLR4 expressed at the surface of immune cells and induce a signal cascade via the nuclear factor kappa B (NFκB) and mitogen-activated protein kinase (MAPK) pathways (Gazzar 2015). It also has a chemotactic role, recruiting monocytes, macrophages, and neutrophils to the site of inflammation and enhancing their adhesion to endothelial cells (Ryckman et al. 2003). It also stimulates leukocyte recruitment and induces cytokine secretion in inflammatory conditions (Wang et al. 2018). Inciarte-Mundo and colleagues showed that higher levels of calprotectin (≥ 3.7 μg/ml) can predict relapse in RA and PsA patients (Inciarte-Mundo et al. 2018). Assuming high levels of calprotectin maintain high secretion levels of pro-inflammatory cytokines including TNFα, we hypothesized that overexpression of calprotectin may limit TNFi efficacy because of a greater TNFα/TNFi ratio. Therefore, TNFi circulating in blood containing high level of calprotectin would not be able to bind all TNFα secreted.

*High mobility group protein B1 (HMGB1)* HMGB1 also known as amphoterin plays a role in autoimmune and auto-inflammatory diseases such as PsA. HMGB1 has been poorly studied in the field of PsA, but high levels have been found in serum and SF of patients with RA and with psoriasis (Taniguchi et al. 2018). High levels of HMGB1 have also been found in patients with type 2 diabetes, obesity and Inflammatory bowel disease (IBD) (Wang et al. 2016; Guzmán-Ruiz et al. 2021; Hu et al. 2015), which are common comorbidities of PsA. These findings suggest that high levels of HMGB1 will also be present in PsA patients. HMGB1 binds to TLR2, TLR4, and RAGE and can then induce synovial inflammation, synthesis of pro-inflammatory cytokines, chemokines, metalloproteinase and adhesion molecules, and cartilage and bone destruction through the NFκB, c-Jun N-terminal kinase (JNK), and p38 signalling pathways. HMGB1 binding can also activate and attract monocytes and neutrophils to sites of inflammation by chemotaxis, and induce proliferation of naïve T cells (Taniguchi et al. 2018; Andersson and Tracey 2011). Moreover, HMGB1 may favour the shift of regulatory T (Treg) cells into the Th17 cell subtype (Papagrigoraki et al. 2017). HMGB1 can either be secreted actively as a cytokine, mainly by macrophages, but also by other immune cells such as monocytes, NK cells, and DCs, or be released passively by necrotic or apoptotic cells (Andersson and Tracey 2011). HMGB1 increases the secretion level of pro-inflammatory cytokines including TNFα, and could limit TNFi efficacy because of a greater TNFα/TNFi ratio, just as for calprotectin. Moreover, by inducing the differentiation of Th17 cells, the levels of pro-inflammatory cytokines such as IL-17A, IL-17F, IL-22, and IL-6 will increase, and their biological activities will not be prevented by TNFi.

##### Advanced glycation end products (AGEs)

Advanced glycation end products are extremely oxidative and reactive compounds formed by a series of chemical reactions. They may also originate from food (when cooked with high dry heat temperatures), UV radiation, and cigarette smoking. AGEs bind to their natural receptor RAGE. The signal involved is not completely known but the binding can induce NFκB, JNK, and p38 pathways and results in the release of pro-inflammatory cytokines such as TNFα, IL-2 IL-4, and IL-1β (Bettiga et al. 2019; Kierdorf and Fritz 2013) and chemokines such as C–C motif ligand (CCL2) (Kierdorf and Fritz 2013). In physiological conditions, AGEs are produced throughout life and accumulate into human tissues and are involved in inflammatory and metabolic disorders. They are overexpressed in patients with hyperglycemia, hyperlipidemia, oxidative stress, and carbonyl stress. The lack of published studies about their involvement in PsA makes it complicated to find if AGEs are also overexpressed in PsA patients. However, obesity, diabetes, and metabolic syndrome are common comorbidities of PsA and are associated with hyperglycemia and hyperlipidemia, and most patients with PsA have a body mass index (BMI) over 25 or 30, indicating overweight or obesity (Husni 2015). As AGEs increase the level of pro-inflammatory cytokines secreted (including TNFα), they could limit TNFi efficacy because of a greater TNFα/TNFi ratio, just as for calprotectin and HMGB1. Therefore, they could be used as a potential biomarker to predict a patient’s response to TNFi.

##### Lymphotoxin α (LTα)

LTα, also known as TNFβ, is another ligand to TNFR. LTα is a homolog of TNFα and possesses the same biological activities (Pennica et al. 1984) (Murdaca et al. 2012). Different immune cells such as CD4 + and CD8 + T cells, B cells, NK cells, and macrophages can express LTα. It also plays a role in the immune system, particularly in the development of lymphoid organs and organization of lymphoid microenvironments, in host defense, and in inflammation (Ware 2005). LTα may promote inflammation through induction of adhesion molecules such as intercellular adhesion molecule (ICAM) and E-selectin in endothelial cells (Pober et al. 1987), or induction of chemokines (Cuff et al. 1998).

**Fig. 3:**
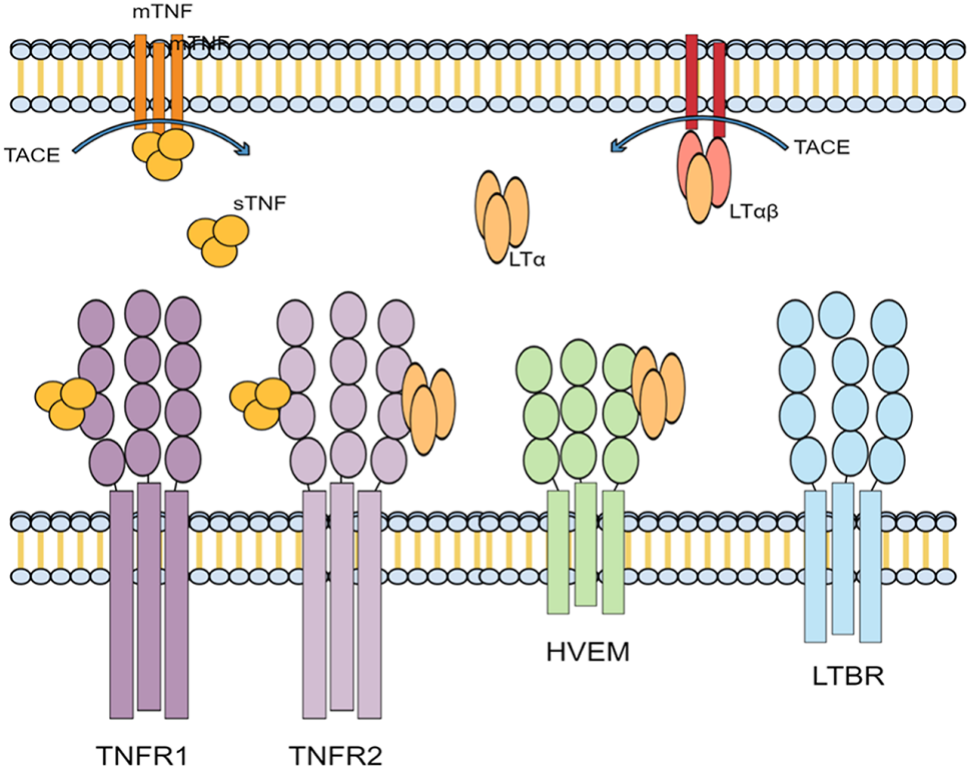
TNF receptors and their ligands. Membrane-bound TNF (mTNF), soluble TNF (sTNF), and LTα can bind to TNF receptor 1 and 2 (TNFR1 and 2). Tumour necrosis factor-alpha converting enzyme (TACE) cleaves mTNF to produce sTNF; it can bind TNFR1 and 2. LTα can be found as a homotrimer and/or in association with membrane-bound LT-β. The homotrimer LTα can bind TNFR1 and the Herpes virus entry mediator (HVEM). Modified from Sedger and McDermott (2014)

Unique among the TNFi used to treat PsA, ETA can bind to LTα whereas INF, ADA, GOL, and CET cannot (Sedger and McDermott 2014). Studies have been conducted to analyse the response of patients to ETA compared to other biologics. Mazzotta and colleagues studied the efficacy of ETA in psoriasis patients after switching from other biologics (Mazzotta et al. 2009). They concluded that patients resistant to INF and efalizumab can respond positively to ETA, but they respond better to ETA if they are biologic naive. Moreover, Conti and colleagues conducted a study on patients with Spondyloarthropathy (SpA), ankylosing spondylitis (AS) and PsA (Conti et al. 2007). They showed that 75% of patients who switched TNFi from INF to ETA responded positively. Based on these studies, we hypothesise that the difference in treatment efficacy could depend on the binding to LTα as this is the main difference between these biologics. Patients with high LTα levels would, thus, respond less to TNFi that cannot bind to LTα. This would make LTα a good candidate biomarker to predict a patient’s response to TNFi and to find an appropriate TNFi (see Fig. 3).

**Fig. 4:**
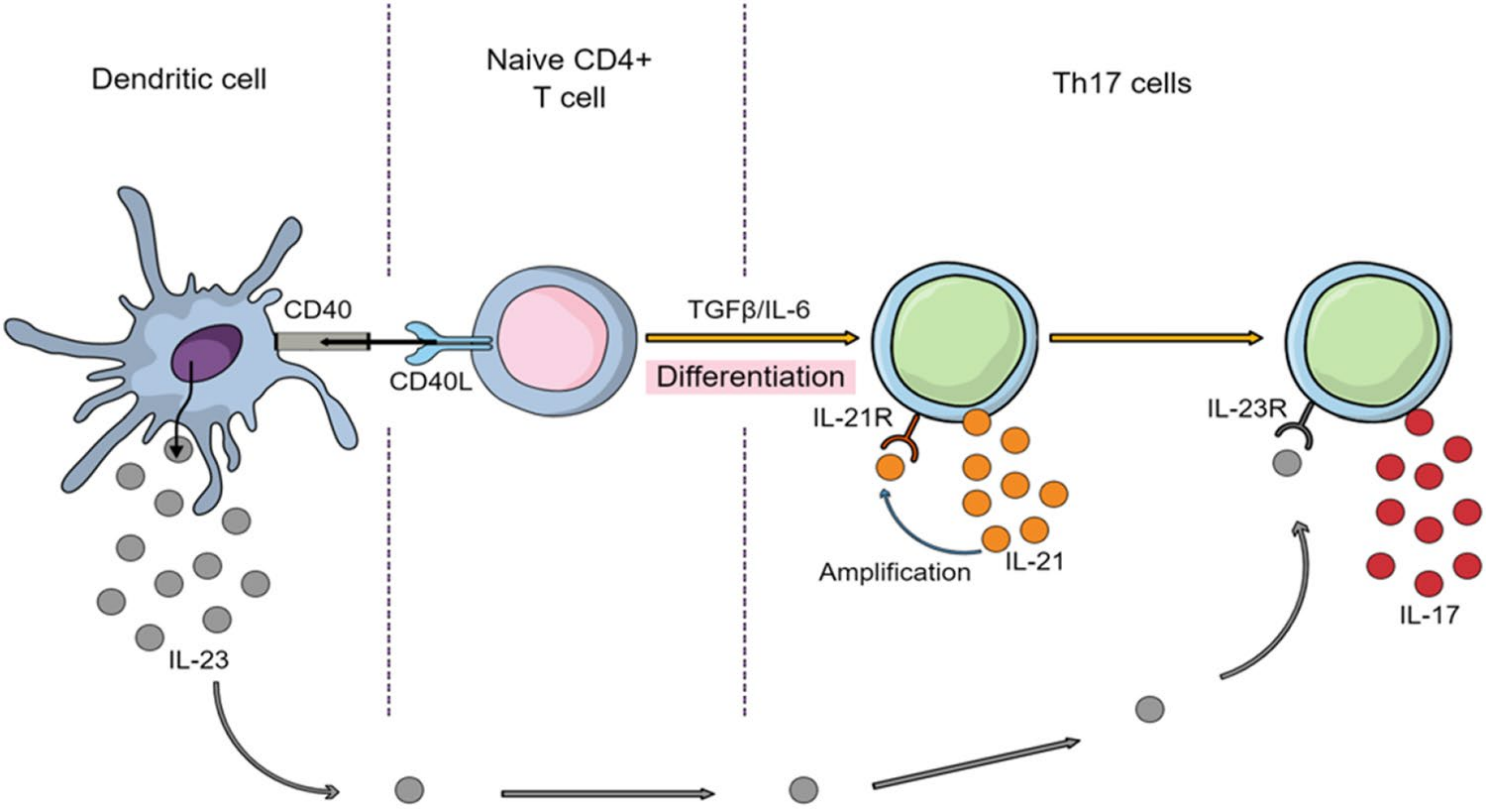
Th17 cells differentiation, amplification, and stabilization. Naïve CD4 + T cells are activated and differentiated into Th17 cells in the presence of TGFβ and IL-6. Th17 secrete IL-17 and IL-21 that amplify Th17 generation in an autocrine manner. IL-21 also induces the IL-23 receptor expression on Th17 cells and makes them responsive to IL-23 stimulation (95). Modified from Murugaiyan and Saha (2009)

Moreover, the LTα gene possesses a DNA-binding site for the HMGB1 protein in its promoter region (Chu 2013). Patients with a high level of LTα may therefore also have a high level of HMGB1 protein, which already plays a role in TNFi resistance as explained above (see Fig. 4).

##### Th17 cells

Th17 cells, and Th17/IL-23 axis in particular, seem of great importance in psoriasis and PsA pathogenesis. Th17 is a subset of CD4 + helper T cells, and IL-23 is an interleukin produced by antigen presenting cells (APCs) that plays a central role in Th17 cell physiology by stimulating differentiation, activation, proliferation, and survival of these cells. IL-23 also stimulates Th17 cells to produce pro-inflammatory cytokines such as IL-17A, IL-17F, IL-6, IL-21, IL-22, and TNF (Bunte and Beikler 2019) (Fig. 4). When patients are treated for PsA, they first receive TNFi and if they fail to respond or become resistant to them, they receive IL-17 inhibitors such as SEC and IXE (Sakkas et al. 2019).

IL-17A targets many cell types such as immune cells (neutrophils, macrophages, monocytes), endothelial cells, fibroblasts, osteoclasts, chondrocytes, osteoblasts, and keratinocytes (Chiricozzi and Krueger 2013). IL-17A induces the secretion of pro-inflammatory cytokines such as the IL-1 family, IL-6, IL-8, and the TNF family (Chiricozzi et al. 2011); and chemokines such as CCL and C-X-C motif ligand (CXCL) families (Harper et al. 2009). Indirectly, IL-17A induces the secretion of angiogenic factors such as vascular endothelial growth factor (VEGF) (Tesmer et al. 2008), adhesion molecules such as ICAM1 (Blauvelt and Chiricozzi 2018), matrix metalloproteinases (MMPs), and receptor activator of nuclear κB ligand (RANKL) (Boyce and Xing 2008) via IL-17 interaction with leukocytes and endothelial cells (Tesmer et al. 2008) (Blauvelt and Chiricozzi 2018). RANKL is involved in osteoclast formation and activation (Boyce and Xing 2008), and, as a consequence, IL-17 can induce and promote joint inflammation and increase cartilage and bone destruction via RANKL expression (Jones et al. 2002).

The level of Th17 cells in blood of PsA patients could be a good marker to predict the response of patients to therapy. Indeed, Miyagawa and colleagues conducted a clinical study in which they classified PsA patients according to their immunological characteristics (Miyagawa et al. 2019). They used phenotyping of peripheral blood CD3 + CD4 + cells to determine which CD4 + cell (Th cells) subset was mostly found in the blood of patients (Th1, Th2 or Th17 cells), and therefore which signalling molecules were most likely involved in the disease’s symptoms. They classified 26 PsA patients into four groups according to which CD3 + CD4 + cell subset was predominant, namely (i) CXCR3 + CCR6-CD38 + HLA-DR + activated Th1 cell-predominant type, (ii) CXCR3-CCR6 + CD38 + HLA-DR + activated Th17 cell-predominant type, (iii) Th1/Th17-high type, and (iv) Th1/Th17-low type. Depending on the group, they administered drugs targeting IL-12/23 (p40) (group i), IL-17 (group ii), TNFα (group iv), and TNFα or IL-17 (group iii). They conclude that disease activity is significantly decreased after 6 months when patients are treated according to their CD3 + CD4 + cell phenotype in all four groups, with no statistical evidence for a difference in response between them. Compared to 38 PsA patients who received standard treatment based on EULAR recommendations, a higher proportion of the 26 patients receiving stratified treatment reached a state of low disease activity after 6 months.

If a high level of Th17 cells is correlated to poor response to TNFi, this might be because the high levels of IL-17 secreted by these cells form the main driver of the inflammatory process. In this case, an IL-17i may be preferable as the first line biologics treatment.

#### Comorbidities as potential biomarkers

PsA is associated with many comorbidities, such as diabetes mellitus, obesity, metabolic syndrome, cardiovascular diseases (CVD), osteoporosis, IBD, autoimmune eye diseases, non-alcoholic fatty liver diseases, kidney diseases, depression, and fibromyalgia (Haddad and Zisman 2017).

Some of the candidate blood biomarkers reviewed above can be overexpressed in patients with these comorbidities. High levels of calprotectin are found in obese patients and in patients with type 2 diabetes, CVD, and IBD (Kruzliak et al. 2014). Likewise, high levels of HMGB1 are found in obese patients (Guzmán-Ruiz et al. 2021) and patients with type 2 diabetes (Wang et al. 2016), CVD, and IBD (Kang et al. 2014). Moreover, adipose tissue can act as an immune organ as it contains many immune cells such as macrophages, T and B cells, neutrophils, eosinophils, and mast cells (Grant and Dixit 2015). Through these, adipose tissue can secrete pro-inflammatory cytokines such as TNFα (Grant and Dixit 2015), and it is also able to secrete alarmins such as HMGB1 (Gunasekaran et al. 2013). AGEs are overexpressed in patients with type 2 diabetes (Vlassara and Striker 2013), liver diseases (Litwinowicz et al. 2021), kidney diseases (Fukami et al. 2015), and in obese patients (Gaens et al. 2013). The presence of one or more comorbidities may further increase the already high levels of these proteins, leading to more severe inflammatory symptoms and a decreased response to TNFi.

In relation to obesity, Th17 cells may be important because obese patients have higher mean levels of circulating IL-17 and IL-23 cytokines than healthy persons (Sumarac-Dumanovic et al. 2009). PsA patients with a BMI over 25 (overweight) or over 30 (obesity) may have higher IL-17 levels, and therefore respond better to IL-17i.

One comorbidity is already used as a biologic stratifier in the latest EULAR guidelines, namely the presence of psoriasis (Gossec et al. 2020). The background to its function as biomarker are the immune cells involved in the development of the skin condition, such as keratinocytes and neutrophils, which secrete IL-17A, IL-22, and IFN-γ (Schön 2019). These pro-inflammatory cytokines are the same cytokines that are secreted by Th17 cells (see potential blood biomarkers above). Miyagawa and colleagues showed that PsA patients with predominantly activated Th17 cells (IL-17 secreting cells) better respond to IL-17i (Miyagawa et al. 2019). PsA patients with severe psoriasis will have a higher level of IL-17 in their blood, and will therefore better respond to IL-17i.

## Discussion/Conclusion

Many candidate biomarkers could be used as potential predictors of response to TNFi and in this review, we classify them into four groups: (1) TNFR-related biomarkers, (2) half-life-related biomarkers, (3) alarmin and inflammation biomarkers, and (4) cell component as a biomarker. Other studies have been undertaken to highlight potential candidate biomarkers able to predict therapeutic response to TNFi (Chandran et al. 2013) (Ademowo et al. 2016) (Hellman et al. 2019), but to date, none of these molecules are used in clinical care (Veale and Fearon 2018) (Winthrop et al. 2019) as they lack specificity. Indeed, it is a challenge to define a response to treatment in patients with PsA as symptoms and features differ from one individual to another. To get around this issue, a combination of biomarkers could be used to verify response to treatment for several conditions such as psoriasis and joint inflammation (Pouw et al. 2020).

First line biologic treatments for PsA patients are TNFi but around 40% of them will not respond to this biologic. These patients will then have to try other treatments to find one that is suitable and effective for them. Biomarkers to predict poor response, easily detectable from a simple blood test, would give them immediate access to more appropriate treatment. Besides saving money, such biomarkers will also shorten the time to achieve low disease activity or remission and improve PsA patients’ quality of life quicker. This review highlights a number of diverse candidate biomarkers that should be investigated for their predictive qualities.

## Data Availability

Enquiries about data availability should be directed to the authors.
